# Effects of Obliterating the Fallopian Tube

**Published:** 1846-03

**Authors:** 


***Effects of obliterating the Fallopian Tube.***—In two female pigs, subjected to experiment in the Veterinary College, the abdomen was opened, as in the common operation of spaying, and the horns of the uterus, together with the ovaria and Fallopian tubes, were freely exposed. In one case, a ligature was placed around the Fallopian tubes, close to the horns of the uterus ; and in the other, a portion of each tube was removed. The operation was similar in effect in both—namely, disconnecting the uterus from the ovaria, the object in view being to ascertain the result upon the desire for sexual intercourse, and the ability of the animal to propagate its species. The pigs were small when operated upon, and both did well, and grew to maturity without exhibiting any symptoms of disease. They were destroyed the day before yesterday, and we find in both instances the Fallopian tubes to be impervious. This state of parts seems not in the least degree to have lessened the desire for copulation, since when the animals arrived at puberty, the labia pudendi became inflamed, with other symptoms denoting œstrum, and the male was taken by both of them. A few weeks after, he was admitted a second time, and, on the animals evincing a desire for the male a third time, they were then slaughtered. In the ovaria, in each, the corpora lutea were distinct, being seemingly about to burst. In one, numerous hydatids were attached to the uterus, while, in the other, large sacs, containing a dark matter, resembling coffee-grounds, were adherent to the ovaria. The parts, also, on cutting into them, appeared unusually vascular, but we must remember, that the animals were destroyed during the existence of sexual excitement. The result of these experiments would seem favourable to the idea, of the passage of the sperma up the Fallopian tubes, in order to impregnate the vesicle of the ovarium; and it certainly proves, that the obliteration of the tubes does not prevent desire, although it renders propagation impossible.—*Mr. Spooner, in Veterinary Transactions* for Nov., as quoted in *Lancet*, 20th Dec. 1844.

